# Evaluation of SINERGIAPS, an intervention to improve patient safety in primary healthcare centers in Spain based on patients’ perceptions and experiences: a protocol for a hybrid type I randomized clinical trial

**DOI:** 10.3389/fpubh.2024.1324940

**Published:** 2024-03-26

**Authors:** Maria A. Fiol-deRoque, Georgina Vidal Mansilla, José A. Maderuelo-Fernández, Olaya Tamayo-Morales, Francisco Martín-Luján, Pilar Astier-Peña, Macarena Chacón-Docampo, Carola Orrego, Montserrat Gens-Barberà, Pilar Andreu-Rodrigo, Ignacio Ricci-Cabello, the SinergiAPS team, Alba Jiménez Mateo, Alexandre Varela Garza, Ana Arceo Tuñe, Ana Belén Ramírez Puerta, Ana Clavería Fontán, Ana Isabel Castaño Carou, Ana María Reales Arroyo, Andrea Rodríguez Covela, Anna Bordas, Anna Mª Ramirez, Antonio Olry de Labry, Arancha García-Iglesias, Aurora Bárbara Martín, Clara Gonzalez Formoso, Cristina Lugones-Sánchez, Encarna Sánchez Freire, Enrique Casado Galindo, Eva Martín, Fernando Álvarez-Guisasola, Fernando Lago Deibe, Helena Vall, Irene Repiso-Gento, Jose Maria Valderas Martinez, Leticia Sierra, Lourdes Luzón Oliver, Luis García-Ortiz, Mari Mar Martínez, Maria de las Nieves Costa Marin, Pablo Lorenzo Rodríguez, Ricardo Rodríguez, Rocío Zamanillo Campos, Sara Maria Guerrero Bernat, Sara Martínez Torres, Tamara Alonso, Yoe Ling, María José Blanco, Maribel Dorado

**Affiliations:** ^1^Health Research Institute of the Balearic Islands (IdISBa), Hospital Universitari Son Espases, Palma, Spain; ^2^Network for Research on Chronicity, Primary Care, and Health Promotion (RICAPPS), Spain; ^3^Unitat de Qualitat i Seguretat dels Pacients, Gerència Territorial Camp de Tarragona, Institut Català de la Salut, Tarragona, Spain; ^4^Unidad de Investigación en Atención Primaria de Salamanca (APISAL), Instituto de Investigación Biomédica de Salamanca (IBSAL), Salamanca, Spain; ^5^Gerencia de Atención Primaria de Salamanca, Gerencia Regional de Salud de Castilla y León (SACyL), Avenida de Portugal, Salamanca, Spain; ^6^Unitat de Suport a la Recerca de Tarragona, Institut de d'investigació en l'Atenció Primària Jordi Gol (IDIAP Jordi Gol), Institut Català de la Salut, Tarragona, Spain; ^7^I-Saude Group, Galicia Sur Health Research Institute (IIS Galicia Sur), Vigo, Spain; ^8^Avedis Donabedian Research Institute (FAD) – Universitat Autonoma de Barcelona, Barcelona, Spain; ^9^CIBER de Epidemiología y Salud Pública (CIBERESP), Madrid, Spain

**Keywords:** patient safety, primary healthcare, randomized controlled trial, avoidable hospital admissions, patient-reported outcome measures

## Abstract

**Background:**

Adverse events in the primary care setting result in a direct cost equivalent to at least 2.5% of total healthcare spending. Across OECD countries, they lead to more than seven million avoidable hospital admissions annually. In this manuscript, we describe the protocol of a trial aimed at evaluating the effectiveness of SinergiAPS (a patient-centered audit and feedback intervention) in reducing avoidable hospital admission and explore the factors that may affect its implementation.

**Methods:**

We will conduct a 24-month, parallel, open-label, multicenter, pragmatic, hybrid type 1 randomized clinical trial. 118 primary healthcare centers with wide geographical distribution in Spain will be randomly assigned (ratio 1:1) to two groups. The intervention group will receive two audits (baseline and intermediate at 12 months) based on information collected through the administration of the PREOS-PC questionnaire (a measure of patient-reported patient safety) to a convenience sample of 100 patients per center. The intervention group will receive reports on the results of both audits, along with educational resources aimed at facilitating the design and implementation of safety improvement plans. The control group will receive care as usual. The primary outcome will be the rate of avoidable hospitalizations (administrative data). Secondary outcomes: patient-reported patient safety experiences and outcomes (PREOS-PC questionnaire); patient safety culture as perceived by professionals (MOSPSC questionnaire); adverse events reported by healthcare professionals (*ad hoc* questionnaire); the number of safety improvement actions which the re has implemented (*ad hoc* questionnaire). Outcome data will be collected at baseline and 24 months follow-up. For the evaluation of the implementation of the SinergiAPS intervention, we will draw on the Consolidated Framework for Implementation Research (CFIR). We will collect and analyze qualitative and quantitative data (30 individual interviews, implementation logbooks; questionnaires for professionals from intervention centers, and level of use of the SinergiAPS web tool).

**Discussion:**

This study will expand the scarce body of evidence existing regarding the effects and implementation of interventions aimed at promoting patient and family engagement in primary healthcare, specifically for enhancing patient safety. The study has the potential to produce an impact on clinical practice, healthcare systems, and population health.

**Clinical Trial Registration**: https://clinicaltrials.gov/study/NCT05958108?term=sinergiAPS&rank=1 (NCT05958108).

## 1 Introduction

Patient safety has been defined as the ability of healthcare systems to minimize the occurrence and impact of unintended or unexpected harm to people during the provision of healthcare (1). It involves efforts to avoid, prevent, and reduce errors, adverse events, and injuries resulting from the provision of healthcare services (2). Adverse events, defined as “*unintended or unexpected incident which causes harm to a patient and may lead to temporary or permanent disability* (3),” can happen in any healthcare setting. While patient safety has traditionally been studied more extensively in hospitals due to the perceived higher risk associated with invasive healthcare procedures, an international systematic review suggests that there are approximately 2–3 patient safety incidents for every 100 primary healthcare care (PHC) consultations, with one out of every 25 incidents causing significant harm to patients (4).

Medical errors are defined as “*an act of omission or commission in planning or execution that contributes or could contribute to an unintended result*” (5). Medical errors related to diagnosis, prescription, and medication usage pose the greatest risk of harm (6). Several key factors contribute to these errors, including the work environment, information transfer between primary and specialized care, the doctor-patient relationship, and ongoing education.

According to a recent OECD report, adverse events in the PHC setting result in a direct cost equivalent to at least 2.5% of total healthcare spending, encompassing additional tests, treatments, and medical care (7). Each year, these events account for over 6% of hospital bed days. Across OECD countries, they lead to more than seven million avoidable hospital admissions annually (7).

The largest epidemiological study of patient safety in Spain (APEAS study, in 2012) found a prevalence of adverse events of 11.2% (IC95%: 10.5–11.9)—of which 64% were considered preventable (8). The cost of these incidents was estimated to reach up to 1 billion euros per year (9), representing approximately 1.6% of the budget of the Spanish National Health System. A more recent epidemiological study in 2020 estimated a prevalence of adverse events of 7.1% 7(95% CI 6.1–8.1%) (10). A national-wide study involving a representative sample of 4,344 healthcare professionals from all regions in Spain showed that patient safety culture as perceived by healthcare professionals is generally positive, with especially higher scores being observed among professionals over 55 years, with managerial responsibilities, women, nurses and administrative staff (11).

### 1.1 Potential of patient experiences and perspectives in enhancing patient safety in PHC

Numerous initiatives and strategies are currently being implemented on both national and international fronts to enhance patient safety and mitigate the prevailing high rates of adverse events. One strategy that is gaining increasing attention is promoting patient participation in their safety (12–14). The World Health Organization’s World Alliance for Patient Safety emphasizes the importance of mobilizing and empowering patients, as evident in their “Patients for Patient Safety” program (15) and the “Global Patient Safety Action Plan 2021–2030” (16). In Spain, enhancing patient involvement in patient safety is also a priority and constitutes one of the six strategic lines of the National Health System’s Patient Safety Strategy 2015–2020 (17).

Patients serve as the common element across various healthcare domains and professions involved in their medical care. As a result, they possess unique insights to evaluate the quality and safety of the healthcare they receive (18). Leveraging this valuable resource, as emphasized can significantly contribute to improving patient safety in PHC. Interventions based on audit and feedback have shown their effectiveness and cost-effectiveness in driving changes in care processes (19) and serve as an excellent framework for developing strategies aimed at engaging patients in safety improvement. These interventions involve gathering patients’ experiences and perceptions of their healthcare through structured questionnaires, processing and transmitting this information to healthcare centers, and utilizing it as a guide to enhance patient safety. The Feedback Intervention Theory (20) underpins these interventions, highlighting that behaviors is influenced by comparing it against standards or goals, and feedback can help identify existing safety issues.

A recent systematic review of the impact of patient and family engagement interventions on patient safety observed positive effects in the reduction of adverse events (13). A recent meta-analysis observed that these types of interventions are beneficial in significantly reducing adverse events (Effect Size = −0.240, *p* < 0.001), decreasing the length of hospital stay (−0.122, p < 0.001), increasing patient safety experiences (ES = 0.630, *p* = 0.007), and improving patient satisfaction (0.268, *p* = 0.004) (21). However, most of the interventions identified up to now have focused on the hospital setting. One of the key challenges in implementing patient involvement strategies in PHC has been the lack of suitable tools for this purpose (12, 13). Patient Reported Outcomes and Experiences Measures (PROMS and PREMS) have emerged as key tools to inform and guide quality and safety improvement initiatives (22). The Patient-Reported Outcomes and Experiences of Safety in Primary Care (PREOS-PC) questionnaire is a tool especially suited for this purpose, originally developed and validated in the United Kingdom (23, 24), and subsequently cross-culturally adapted, translated, and validated in multiple languages, including Brazilian (25), and Spanish and Catalan (26).

### 1.2 The SinergiAPS intervention

Our team has contributed to the field by developing and evaluating the SinergiAPS (“Synergies between Professionals and Patients for Safe Primary Care”) intervention (27). This intervention aims to enhance patient safety in PHC by incorporating the perspectives and experiences of the patients themselves. In designing this intervention, a diverse range of stakeholders, including patients (28) and healthcare professionals (29), were actively involved. SinergiAPS is grounded in the Clinical Performance Intervention Theory (30) and was developed following the guidelines for complex intervention development proposed by the Medical Research Council. A detailed description of the SinergiAPS intervention is available below.

SinergiAPS was developed and evaluated as part of the project “Development and Evaluation of an Intervention based on Patient-Provided Feedback to Improve Patient Safety in Primary Care Centers” (27). The intervention underwent a 12-month follow-up randomized clinical trial involving 9,668 patients and 1,053 professionals from 59 PHC centers in Spain. Unfortunately, the trial was significantly impacted by the outbreak of the COVID-19 pandemic. Findings from a post-trial qualitative study (in-depth interviews with 14 professionals from the intervention group centers), revealed that the intervention could not be implemented as originally planned due to the exceptional circumstances faced by the participating centers (31). In addition to the challenges posed by the pandemic, we identified potential barriers to the large-scale implementation of SinergiAPS intervention as part of routine clinical practice: the face-to-face administration of PREOS-PC questionnaires was resource-intensive; healthcare professionals lacked time during regular working hours to analyze patient feedback reports; insufficient knowledge about how to design safety improvement actions, and; scarcity of resources to implement them.

### 1.3 Study justification

Initiatives to improve patient safety have traditionally focused on information supplied by healthcare professionals, ignoring the views of the patients themselves (32). In recent years, there has been a substantial surge in interventions grounded in the active engagement of patients and their families to enhance patient safety, as evidenced by a recent systematic review (13). However, most of these interventions have thus far emanated from hospital environments (33). A small number of studies have evaluated patient feedback interventions to improve safety in the PHC setting, but they are limited by the fact that are pilot studies with small sample sizes and short follow-up periods (14, 34).

The SinergiAPS intervention has already shown promise in reducing avoidable hospitalizations in a previous pilot study, as well as a high level of acceptability and perceived utility among patients PHC teams, and health managers. However, before its widespread implementation in PHC centers, it is imperative to obtain solid empirical evidence about its effectiveness and implementation through a large and robust randomized clinical trial.

### 1.4 Aims

In this manuscript, we report the details of the protocol of a trial aimed at evaluating the effectiveness and implementation of SinergiAPS, an intervention based on patient perceptions and experiences, aimed at improving patient safety in PHC centers. The specific objectives of the trial are:

1. To evaluate the effectiveness of the SinergiAPS intervention in improving patient safety in PHC centers by reducing avoidable hospitalizations (primary outcome variable), as well as increasing the safety culture, increasing the number of patient safety improvement actions in the centers, and enhancing patient-perceived safety (secondary outcome variables).
2. to study the implementation of the SinergiAPS intervention in PHC centers in Spain (with the ultimate goal of offering a framework that could be implemented in other health systems worldwide), by: (i) determining the level of usage of the intervention (both of whole the intervention and of each of its constituents); (ii) identifying barriers and facilitators for the implementation of the intervention (including factors related to the intervention itself; to the inner characteristics of the PHC centers; or to the external (contextual) environment within they operate), and; (iii) understanding the processes by which the intervention is successfully implemented.

## 2 Methods and analysis

### 2.1 Design

Hybrid type 1 clinical trial, pragmatic, multicenter, open-label, with a 24-month follow-up. It has been designed according to the CONSORT statement (35). This protocol has been drafted following the SPIRIT guidelines for randomized trials (36) (Supplementary file 1). It has been registered on ClinicalTrial.gov in August 2023 (NCT05958108).

**Figure 1:**
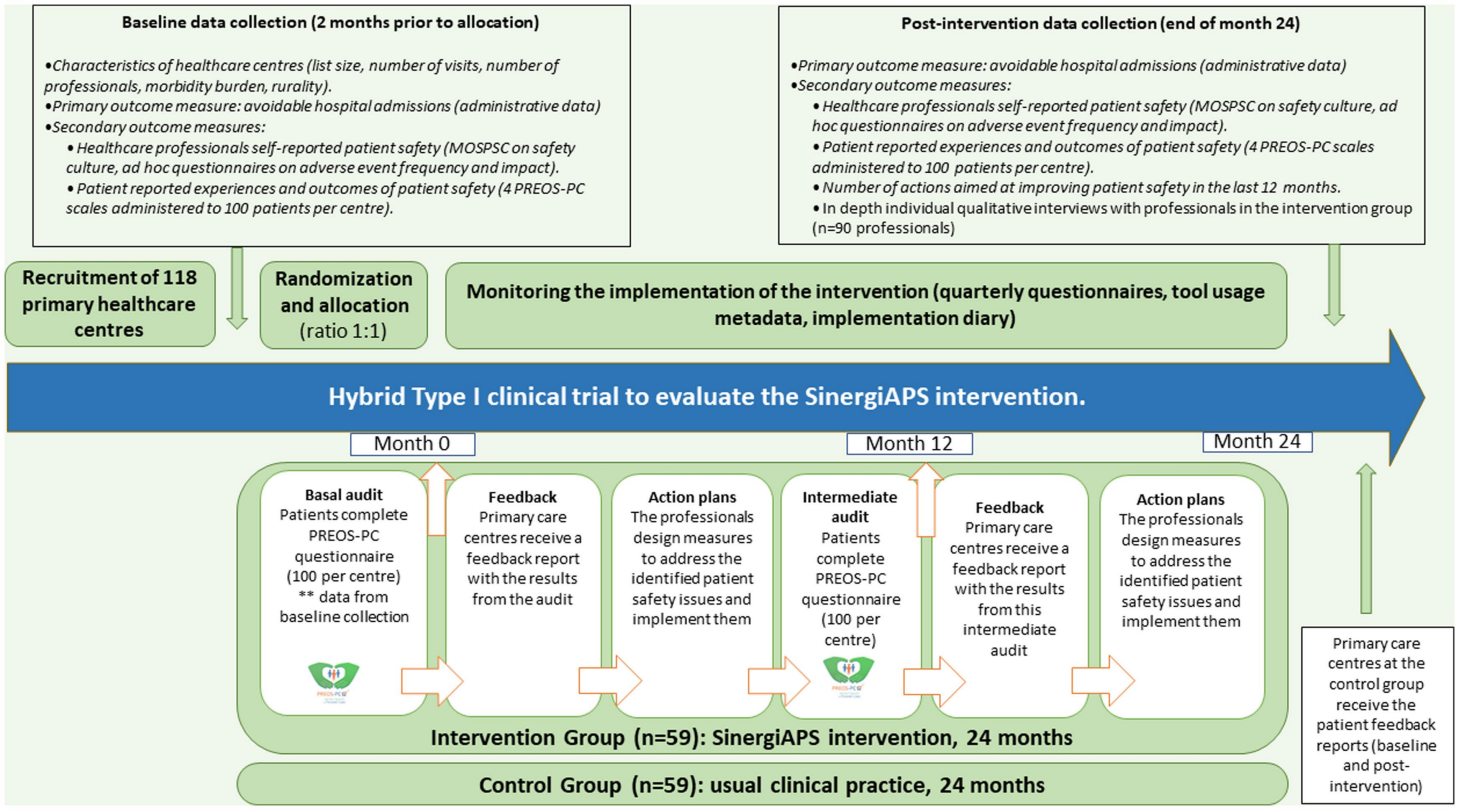
Flow diagram of the Type I hybrid clinical trial to evaluate the SinergiAPS intervention.

In this trial, the PHC centers will be randomly assigned to the intervention group (which will receive the SinergiAPS intervention—described below) and the control group (waitlist design: usual clinical practice during the 24-month follow-up, after which they will have access to the SinergiAPS intervention). Figure 1 provides an overview of the trial, including the schedule of enrolment, interventions, and assessments.

### 2.2 Description of the SinergiAPS intervention

**Figure 2:**
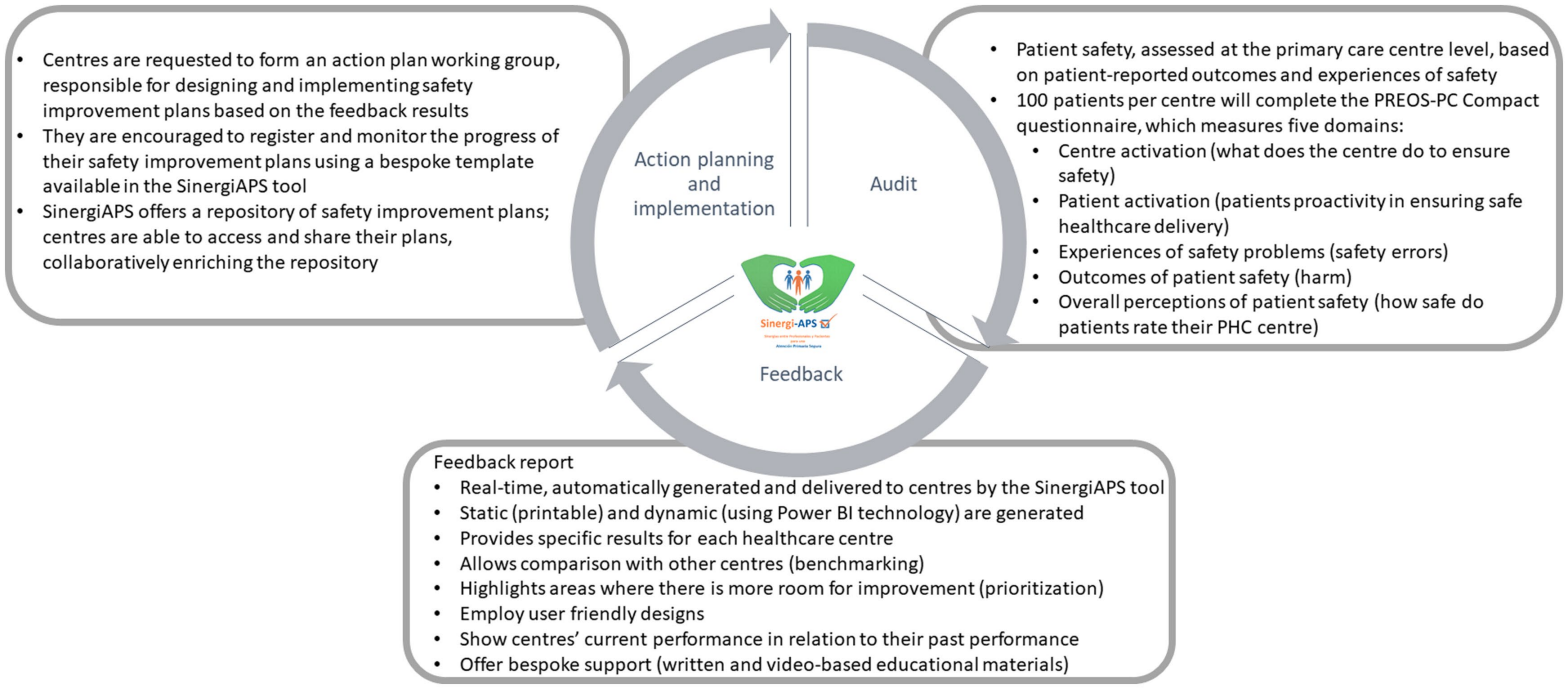
Description of the SinergiAPS intervention.

SinergiAPS is a tool designed to support PHC centers to identify potential problems and areas for improvement related to patient safety based on information provided by their own patients. This intervention consists of three main elements (Figure 2):

- Patient safety audit of PHC centers: The patient safety of all the PHC received during the previous 12 months is evaluated from the perspective of patients using the validated Patient Reported Experiences and Outcomes of Safety in Primary Care (PREOS-PC) Compact questionnaire. A baseline audit will be conducted at the beginning of the intervention, followed by an intermediate audit at 12 months.
- Feedback report of results to centers: SinergiAPS automatically generates near-real-time feedback reports with the audit results. This report is specific to each center, and it includes a comparison with the rest participating centers to facilitate benchmarking. Centers are encouraged to form a working group, consisting of approximately 3–6 professionals with a designated leader.
- Design of action plans: The working group of each center convenes to design patient safety improvement plans based on the problems identified in their center’s result report. For this purpose, the SinergiAPS web tool provides resources, training materials, and recommendations on how to enhance patient safety in PHC. Additionally, it offers a tracking template to collect and evaluate the measures proposed by the centers to address the identified safety issues, as well as a repository of action plans designed and implemented by other healthcare centers.

### 2.3 Participating healthcare centers and sampling

The study units in this trial are public PHC centers from any autonomous community in Spain that agree to participate through informed consent (Supplementary file 2). Centers that exclusively provide specific services (such as pediatrics or women’s health centers), and centers that have been established for less than 12 months at the time of recruitment will be excluded.

Recruiting PHC centers: We will recruit PHC centers from various regions in Spain. Each participating node will recruit centers from their own community and, whenever possible, from neighboring regions. To ensure a broad geographic representation, researchers from seven regions (Balearic Islands, Catalonia, Andalusia, Madrid, Castilla y León, Aragon, and Galicia) are involved in this multicenter study. In regions where we do not have a specific node or researcher, we will leverage our extensive network of key informants, including researchers from the Patient Safety Group of SEMFYC, the research boards of SEMFYC, and the national network of the European General Practice Research Network. This established network will significantly aid in identifying and engaging suitable healthcare centers for participation in this study. The study proposal will be presented to the relevant territorial management authorities and healthcare centers to garner support and participation. A healthcare center will be considered recruited once informed consent is obtained from the center coordinator.

Sampling of PHC centers: For each region, an intentional sample of centers will be selected, aiming for heterogeneity in terms of size, rurality, and inclusion of both teaching and non-teaching centers.

### 2.4 Participating PHC professionals and patients

Selection of PHC professionals: We will include all the professionals (both healthcare and non-healthcare personnel) working in the healthcare centers recruited. We will exclude those who have been working at the center for less than 3 months from the time of the invitation. Invitations along with questionnaire links (detailed in the “outcome variables”) will be sent to professionals via email. The questionnaires will be self-administered electronically for ease and convenience.

Selection of patients: Using our information systems, we will randomly select patients meeting the following eligibility criteria: aged 18 years or older; registered at each participating healthcare center; having visited (either in-person or remote) their healthcare centers for a health-related issue within the last 3 months (to minimize recall bias). We will exclude patients not able to understand Spanish, Catalan or Galician. These patients will be invited to self-complete the validated PREOS-PC Compact questionnaire over the phone or via email. In addition, patients may also be invited to complete the questionnaire face-to-face in the center waiting room. In such cases, a research assistant will approach patients consecutively and invite those meeting the eligibility criteria described above to self-complete the questionnaire using tablet computers or their smartphones.

### 2.5 Data collection

#### 2.5.1 PHC center characteristics

We will collect the following information for each center (Table 1): total number of visits in the last 12 months, total count of registered patients at the center, the patient-to-physician ratio, the percentage of patients aged over 65 years, the percentage of female patients, the academic affiliation (whether the center is a teaching center or not), the rurality index (37), deprivation index (38), and the distribution of patients according to adjusted morbidity group (39). This data will be sourced from healthcare information systems of the health services from the participating regions.

**Table 1:** Data collection schedule for the SinergiAPS 2 trial.

	Source	Aggregation level	Data collection timing
1. Characteristics of the primary healthcare centers (all recruited centers)			
Number of visits to the center in the last 12 months	Administrative data	Practice level	Baseline
List size (total number of registered patients at the center)	Administrative data	Practice level	Baseline
Average number of patients per physician in the center	Administrative data	Practice level	Baseline
Percentage of patients over 65 years old in the center	Administrative data	Practice level	Baseline
Percentage of female patients	Administrative data	Practice level	Baseline
Teaching center (yes/no)	Administrative data	Practice level	Baseline
Rurality index	Administrative data	Practice level	Baseline
Adjusted morbidity group (GMAs—percentage of patients in each of the five complexity groups)	Administrative data	Practice level	Baseline
2. Effectiveness evaluation (all recruited centers)			
Rate of avoidable hospitalizations during the previous 12 months (primary outcome measure)	Administrative data	Practice level (number hospitalizations/10000 registered patients)	Baseline and 24 months follow-up
Activation of healthcare centers: 4 items (Cronbach’s α = 0.70) that measure the proactivity of healthcare centers towards patient safety.	Patient-reported (PREOS-PC Compact)	Practice level (mean score—theoretical range: 0 to 100)	Baseline and 24 months follow-up
Experience of harm (severity): 3 items (Cronbach’s α = 0.85) that assess the level of harm suffered by patients as a result of the healthcare they received.	Patient-reported (PREOS-PC Compact)	Practice level (mean score—theoretical range: 0 to 100)	Baseline and 24 months follow-up
Burden of harm: 3 items (Cronbach’s α = 0.73) that evaluate the impact and consequences of the harm experienced by patients	Patient-reported (PREOS-PC Compact)	Practice level (mean score—theoretical range: 0 to 100)	Baseline and 24 months follow-up
Overall assessment of the center’s safety level (1 item). Visual analogue scale.	Patient-reported (PREOS-PC Compact)	Practice level (mean score—theoretical range: 0 to 100)	Baseline and 24 months follow-up
Patient safety culture	Healthcare provider-reported (MOSPSC questionnaire)	Practice level (mean score—theoretical range: 0 to 5)	Baseline and 24 months follow-up
Frequency of adverse events experienced by healthcare professionals in the last 12 months	Healthcare provider-reported (*ad hoc* questionnaire)	Practice level (mean score—theoretical range: 0 to 100)	Baseline and 24 months follow-up
Impact of adverse events on healthcare providers wellbeing	Healthcare provider-reported (*ad hoc* questionnaire)	Practice level (mean score—theoretical range: 0 to 100)	Baseline and 24 months follow-up
Number of actions aimed at improving patient safety in the center in the last 12 months	Reported by the practice responsible for patient safety	Practice level (total number)	24 months follow-up
3. Implementation and process evaluation (only centers allocated to the intervention group)			
Number of accesses to the SinergiAPS tool	Metadata from SinergiAPS tool	Practice level (total number)	Monthly during the 24 months of follow up period
Time using the SinergiAPS tool	Metadata from SinergiAPS tool	Practice level (number of minutes)	Monthly during the 24 months of follow up period
Number of safety improvement actions registered in the SinergiAPS tool	Metadata from SinergiAPS tool	Practice level (total number)	Quarterly during the 24 months of follow up period
Usage of the SinergiAPS tool	Reported by the practice responsible for patient safety (*ad hoc* questionnaire)	Practice level (number of hours)	Quarterly during the 24 months of follow up period
Perceived utility and satisfaction with the SinergiAPS tool	Reported by the practice responsible for patient safety (*ad hoc* structured questionnaire)	Practice level (mean score—theoretical range: 0 to 100)	Quarterly during the 24 months of follow up period
Overall assessment of the SinergiAPS intervention, identification of aspects for improvement, and of suggestions to facilitate the large-scale implementation of SinergiAPS	In depth qualitative interviews with health-care professionals	Regional level (narrative experiences)	After the 24 months follow up
Implementation barriers	Researchers in from each region (implementation diary)	Regional level (narrative experiences)	Continuously during the 24 months follow up

MOSPSC, Medical Office Survey on Patient Safety Culture.

#### 2.5.2 PHC professional’s characteristics

We will collect the following characteristics from the healthcare professionals: age, gender, time worked in PHC, time worked at the center, number of work hours per week, professional category, number of assigned patients to their list, employment status (permanent, temporary), work shift (morning, afternoon, both), participation in on-call duties, perceived health status, job satisfaction on a scale from 0 to 10.

#### 2.5.3 Patient characteristics

Age, gender, educational level, nationality, employment status, number of visits to the PHC center in the last 12 months, duration of registration at the center, self-perceived health status, number of medications, and presence of chronic diseases.

### 2.6 Sample size

The sample size calculation is based on the primary outcome variable of this trial, the rate of avoidable hospitalizations. To achieve a statistical power of 80% with an alpha risk of 0.05 in a one-sided test, a total of 118 healthcare centers (59 in the intervention group and 59 in the control group) are required to detect a difference equal to or greater than 1.5 units in the rate of avoidable hospitalizations per 1,000 patients, corresponding to a 15% reduction in the rate of avoidable hospitalizations (Cohen’s d = 0.49). Based on the results of our previous study (31), we assume a mean rate of 11.2 and a standard deviation of 3.3. A follow-up loss rate of 0.5% has been estimated.

### 2.7 Randomization of PHC centers

After recruiting the centers and collecting baseline data, they will be randomly assigned to either the control group or the intervention group in a 1:1 ratio. This randomization process will be performed using specialized software that generates a list of random numbers by a statistician not otherwise involved in the study data collection. Blinding of center allocation to the PHC professionals will not be possible due to the characteristics of the intervention. To ensure balance and representation, we will apply stratified randomization, a well-established approach to balance one or a few prespecified prognostic characteristics between treatment groups (40, 41). We identified two prognostic characteristics that could be strongly associated with the primary outcome of the trial: the healthcare region the PHC belonged to (which determines the intensity and types of safety improvement initiatives routinely conducted by regional healthcare managers), and being or not an accredited PHC teaching center (as teaching centers are frequently more easily engaged in improvement activities). To help ensure the balance of treatments within strata and the balance of strata within treatment groups, the assignment procedure within each stratum will be restricted using permuted blocks (41).

### 2.8 Evaluation of the effectiveness of the SinergiAPS intervention

Our first objective is to evaluate the effectiveness of the intervention at 24 months in improving the safety of the healthcare provided in the PHC centers. We will examine the impact of the intervention on the following outcome measures:

#### 2.8.1 Primary outcome measure

The rate of avoidable hospitalizations will be assessed based on the definition provided by the Agency for Health Research and Quality, based on data extracted from the Minimum Basic Data Set using predefined ICD-9 codes (42). The rate of avoidable hospitalizations will be calculated specifically for conditions such as asthma, chronic obstructive pulmonary disease, congestive heart failure, angina, diabetes, and chronic kidney disease. For each participating center, we will obtain the total number of registered patients and the total number of avoidable hospitalizations recorded during the previous 12 months.

#### 2.8.2 Secondary outcome measures

1. Perceptions and experiences of patients (scores from the scales of the PREOS-PC Compact questionnaire): This questionnaire will be administered to a selected sample of 100 patients from each healthcare center. This sample size is estimated to achieve a reliability of at least 0.7 in the mean scores of the healthcare centers for the questionnaire’s scales. The questionnaire incorporates the following scales:

- “Practice activation”: 4 items (Cronbach’s α = 0.71) that measure the proactivity of healthcare centers towards patient safety.
- “Experience of harm (severity)”: 3 items (Cronbach’s α = 0.81) that assess the level of harm suffered by patients because of the healthcare they received.
- “Burden of harm”: 3 items (Cronbach’s α = 0.80) that evaluate the impact and consequences of the harm experienced by patients.
- “Overall assessment of the center’s safety level” (1 item). Visual analogue scale.

1. Patient safety culture of healthcare professionals: We will calculate the average center-level score of the Patient Safety Culture Composite Score (ranging from 1–5 points), based on the responses of professionals to the Spanish version of the Medical Office Survey on Patient Safety Culture (MOSPSC) questionnaire (39).
2. Adverse events experienced by healthcare professionals in the last 12 months: An ad hoc questionnaire will be adapted from a previous study (43) to assess the adverse events experienced by professionals (frequency and impact on healthcare professionals’ well-being).
3. Number of actions aimed at improving patient safety in the center in the last 12 months: An ad hoc questionnaire will be administered to the quality and patient safety referent of each center, including both intervention and control centers, to determine the number of actions taken to enhance patient safety.

#### 2.8.3 Statistical analysis

Initially, we will conduct descriptive statistical analysis to characterize the participating healthcare centers. For each of the two groups (control and intervention), we will calculate frequencies (percentages) for binary or categorical variables, and medians (along with interquartile range) for continuous variables. The effectiveness of the intervention will be analyzed by comparing the mean score of the primary outcome variable (rate of avoidable hospitalizations) between the control and intervention groups after 24 months of follow-up. A linear regression model will be used, including the baseline rate as an adjusting variable. Moreover, stratified effectiveness analyses will be performed based on baseline audit scores. This approach will enable us to measure the independent effect of the intervention in centers with different levels of potential improvement according to the audit results. All analyses will be conducted on an intention-to-treat basis by a statistician blinded to group allocation, ensuring that participants are analyzed according to their assigned group irrespective of any deviations from the intervention. If necessary, we will handle missing data with multiple imputation. An equivalent methodology will be applied for the secondary outcome variables. All outcome variables (including scores from patient and healthcare professional questionnaires) will be analyzed at the level of healthcare centers (mean score per center). Regarding PREOS-PC scores, we will explore the extent to which the different methods of administration of the questionnaire (face-to-face, telephone, or by email) could yield systematic differences in the scores obtained. In case they do, to minimize a potential confounding bias, we will include the method of administration as an adjustment variable in our regression model. All analyses will be carried out in Stata 14.1 (Stata Corp), using an α of 5% throughout.

### 2.9 Evaluation of the implementation of the SinergiAPS intervention

Our second objective is to assess the level of success in the implementation of the SinergiAPS intervention and identify contextual factors associated with greater implementation success. This will provide the necessary evidence to design a future large-scale implementation strategy for SinergiAPS. To achieve these goals, we will evaluate each of the five constructs of the Consolidated Framework for Implementation Research (CFIR) model (44): the intervention itself, the inner setting, the outer setting, the individuals involved, and the processes by which the intervention implementation is achieved.

#### 2.9.1 Data collection

- In-depth qualitative interviews with professionals from the intervention group (approximately 30 qualitative interviews, until reaching saturation of discourse). These interviews aim to examine the overall assessment of the SinergiAPS intervention, identify aspects for improvement, and gather suggestions to facilitate the large-scale implementation of the intervention. Sampling will be intentional, including informants of both genders, different age groups, professional categories, and territorial areas.
- Quantitative interviews: Through an online questionnaire administered quarterly to the project referent in each center, we will measure the usability of the intervention, as well as the degree of use, acceptability, and perceived usefulness.
- Level of usage of the SinergiAPS web tool: We will examine the extent of the tool’s utilization by monitoring the number of accesses made by healthcare centers. We will determine whether the healthcare centers have accessed the feedback report or not. Additionally, we will determine the frequency of accesses and usage time of the module within the tool that provides training and support materials for designing and implementing action plans.
- Analysis of the action plans proposed by the healthcare centers: The web tool includes a module aimed at systematically recording the action plans formulated by healthcare centers. This comprehensive repository of information remains securely registered within the tool and provides valuable insights for examining the implementation of the intervention.
- Implementation diary: In each node, the researchers will maintain an active record of the barriers encountered during the implementation process of the intervention in the centers.

#### 2.9.2 Data analysis

We will analyze the quantitative data (quantitative interviews and level of usage of the SinergiaAPS tool) using descriptive statistics at the center level (e.g., mean and standard deviation of usability and perceived utility scores). We will conduct a trend analysis to monitor the levels of usage over time, every 3 months.

We will analyze the qualitative data from the in-depth qualitative interviews with professionals using thematic analysis (45). For the analysis of the action plans proposed by the healthcare centers, and of the implementation diary, we will use content analysis (46). Both types of analysis will be conducted independently by two experienced researchers. Discrepancies will be discussed with the research team until an agreement is reached concerning the list of themes and codes. We will use NVivo 14 to support the analysis of the qualitative data.

### 2.10 Trial management

The main coordinating center will be at the Primary Care Research Unit of Mallorca—the Balearic Islands Health Research Institute (IdISBa). A trial coordinator from each participant region will contact PHC center coordinators to manage recruitment, trial initiation, and implementation of the intervention as well as for the qualitative data collection for the process evaluation. They will also be responsible for collecting the data from the included centers, professionals and patients. Patient and professional identifiable data will be accessible only to the researchers from the same region. We will generate a database with no identifiable information, which will be accessed by the trial analyst (blinded to group allocation) through a password-protected portal. Data from implementation analysis will also be kept in a password-protected portal.

Trial modifications will undergo review by the Ethics Committee for approval, will be documented in our registered protocol, and will be included in the final manuscripts.

## 3 Discussion

The proposed study has the potential to have an impact on clinical practice, healthcare systems, and population health. In terms of clinical practice, this study will provide the evidence-based tool, SinergiAPS, which enables PHC centers in Spain to systematically collect patients’ perceptions and experiences concerning the safety of their care. Moreover, it will offer a structured approach for the centers to receive and integrate this information with other patient safety intelligence, enabling the development of patient-centered action plans for specific improvements. In terms of impact on the healthcare system, the proposed SinergiAPS intervention has been designed to minimize costs while maximizing scalability and sustainability. By utilizing a dedicated web-based tool for collecting patient experiences and automatically generating customized feedback reports, it can be widely implemented in PHC centers with limited external support and at a low cost. As such, this intervention has the potential to be a cost-effective, feasible, and sustainable strategy for enhancing patient safety in the PHC setting. If effective, it could contribute to reducing the estimated annual costs associated with safety incidents in PHC [around 2.5% of total healthcare spending (7)].

In terms of impact on population health, it is worth noting that experiencing errors in the PHC settings can lead to severe harm and even death. Notably, a recent OECD report highlights adverse events as the cause of over 7 million avoidable hospitalizations each year (7). Such incidents also result in emotional distress for patients and healthcare professionals, with the latter considered second victims of adverse events. The widespread implementation of the SinergiAPS tool in PHC centers have the potential to play a significant role in preventing harm, reducing avoidable hospitalizations, and improving population health. Notably, patient safety issues in PHC disproportionately affect socially vulnerable groups, such as women and individuals with lower educational levels, as evidenced by recent research (47). Therefore, if SinergiAPS proves effective, it could contribute to reducing health inequalities.

### 3.1 Strengths and limitations of the proposed study

This study represents the first large-scale trial to evaluate an intervention to improve patient safety in PHC centers through the integration of patient-reported safety experiences and outcomes. By involving a significant number of centers, this research aims to comprehensively assess the impact of the intervention over a 24-month follow-up period, using a combination of consolidated administrative data, such as avoidable hospital admissions, as well as validated questionnaires like PREOS-PC and MOSPSC. The evaluation of the implementation of SinergiAPS will be guided by CFIR, a well-established and widely recognized framework. By adopting CFIR, we will be able to holistically examine the factors influencing the implementation process, considering both internal and external elements that may affect the intervention’s success. All these are novel aspects that make our study unique in terms of study design and approach.

The study also has some limitations that warrant consideration. Firstly, being a multicenter project with researchers from several regions in Spain and a comprehensive network of national collaborators, recruiting centers from all regions may pose challenges. Nevertheless, this would have a minimal effect on the overall number of healthcare centers to be recruited, which remains manageable within the participating nodes. Although this limitation is not expected to significantly impact the external validity of the study, the exclusion of centers from specific regions could restrict the evaluation of SinergiAPS implementation due to the unique characteristics of regional healthcare systems. Secondly, intentional sampling of healthcare centers has been chosen as the preferred sampling method, as random sampling is not feasible and could introduce potential selection bias. Thirdly, patient safety research in PHC is still in its early stages, and the lack of established “gold standard” measures for evaluating patient safety could constrain the comprehensive assessment of SinergiAPS’s effectiveness. However, the proposed outcome variables collectively encompass a wide range of aspects related to patient safety in PHC, allowing for evaluation from multiple perspectives. Lastly, due to the nature of the intervention, blinding of healthcare centers is not possible. However, randomization will be conducted after baseline data collection, and efforts will be made to maintain blinding among analysts to minimize bias. It will be crucial to address this potential source of bias in the analysis and interpretation of the study results.

## 4 Conclusion

This study holds the potential to make a substantial contribution to the current body of evidence regarding the effects of interventions aimed at promoting patient and family engagement in PHC, particularly in the context of enhancing patient safety. If effective, this highly scalable and low-cost intervention could be rolled out for its use as part of routine clinical practice—potentially leading to a decrease in adverse events and avoidable hospital admissions in Spain.

## Ethics statement

The studies involving humans were approved by the Balearic Islands Ethics Committee (IB 5144/23 PI). The studies were conducted in accordance with the local legislation and institutional requirements. The participants provided their written informed consent to participate in this study.

## Author contributions

MF-d: Writing – original draft, Conceptualization. GM: Writing – review & editing. JM-F: Writing – review & editing, Methodology, Funding acquisition. OT-M: Writing – review & editing. FM-L: Writing – review & editing, Writing – original draft, Funding acquisition. PA-P: Writing – original draft, Funding acquisition. MC-D: Writing – review & editing. CO: Writing – review & editing, Writing – original draft, Funding acquisition, Conceptualization. MG-B: Writing – review & editing, Funding acquisition. PA-R: Writing – review & editing. IR-C: Writing – original draft, Methodology, Funding acquisition, Conceptualization.

## The SinergiAPS team

Alba Jiménez Mateo; Alexandre Varela Garza; Ana Arceo Tuñe; Ana Belén Ramírez Puerta; Ana Clavería Fontán; Ana Isabel Castaño Carou; Ana María Reales Arroyo; Andrea Rodríguez Covela; Anna Bordas; Anna Mª Ramirez; Antonio Olry de Labry; Arancha García-Iglesias; Aurora Bárbara Martín; Clara Gonzalez Formoso; Cristina Lugones-Sánchez; Encarna Sánchez Freire; Enrique Casado Galindo; Eva Martín; Fernando Álvarez-Guisasola; Fernando Lago Deibe; Helena Vall; Irene Repiso-Gento; Jose Maria Valderas Martinez; Leticia Sierra; Lourdes Luzón Oliver; Luis García-Ortiz; Mari Mar Martínez; Maria de las Nieves Costa Marin; Pablo Lorenzo Rodríguez; Ricardo Rodríguez; Rocío Zamanillo Campos; Sara Maria Guerrero Bernat; Sara Martínez Torres; Tamara Alonso; Yoe Ling; María José Blanco; Maribel Dorado.
