# Supplementary material for: Evaluation of SINERGIAPS, an intervention to improve patient safety in primary healthcare centers in Spain based on patients’ perceptions and experiences: a protocol for a hybrid type I randomized clinical trial
Source: Front Public Health. 2024 Mar 26;12:1324940. doi: 10.3389/fpubh.2024.1324940 (PMC11003190; doi:10.3389/fpubh.2024.1324940)
Supplement: Supplementary file 2 [file Data_Sheet_2.PDF]

**Additional file 2. Model consent form for Primary Healthcare Centres**

**CONSENTIMIENTO INFORMADO**

TÍTULO DEL ESTUDIO: Evaluación de SINERGIAPS, una intervención dirigida a mejorar la seguridad del paciente en centros de salud en España en base a las percepciones y experiencias de los propios pacientes: Ensayo clínico híbrido tipo I
CÓDIGO DEL PROMOTOR: PI22/01521
PROMOTOR: Instituto de Salud Carlos III (Ministerio de Ciencia e Innovación)
INVESTIGADOR PRINCIPAL: Ignacio Ricci Cabello (971 175883). Investigador Titular. Grupo de Investigación en Atención Primaria y Promoción -Comunidad Autónoma de las Islas Baleares (GRAPP-caIB) del Institut d`Investigació Sanitària Illes Balears
CENTRO DE TRABAJO: Gerencia de Atención Primaria de Mallorca (GAP). Carrer de l'Escola Graduada, 3 07002 Palma, Illes Balears.

Nombre del centro de salud: ...............................

Nombre y apellidos del coordinador/a del Centro de Salud:...............................

☐ He podido leer la hoja de información sobre este estudio.

☐ He recibido suficiente información sobre el estudio.

☐ He obtenido toda la información relativa al tratamiento de datos del centro.

☐ He podido hablar con alguna de las personas responsables de este estudio en caso de así desearlo.

☐ Comprendo que la participación del centro es voluntaria.

☐ Comprendo que el centro puede retirarse del estudio:

- Cuando quiera.
- Sin tener que dar explicaciones.
- Sin que esto tenga ningún tipo de repercusión negativa.

☐ Comprendo que, si decido retirar el centro del estudio, los resultados obtenidos hasta ese momento podrán seguir siendo utilizados.

En el caso de que los resultados de la investigación proporcionen datos que me puedan interesar: (*indicar una de las casillas*)

☐ Quiero ser informado.

☐ No quiero ser informado

☐ **COMO COORDINADOR DEL CENTRO, PRESTO LA CONFORMIDAD PARA QUE EL CENTRO PUEDA PARTICIPAR EN EL ESTUDIO**

[Firma del coordinador]

Nombre: Nombre: Ignacio Ricci Cabello
Fecha: Fecha:
